# Knowledge, attitudes and practices of sharps waste disposal by diabetic patients in rural South Africa

**DOI:** 10.4102/safp.v65i1.5538

**Published:** 2023-01-10

**Authors:** Lihle Ziqubu, Dudu G. Sokhela, Sibusiso D. Gabela

**Affiliations:** 1 Department of Community Health Studies, Faculty of Health Sciences, Durban University of Technology, Durban, South Africa; 2 Department of Nursing, Faculty of Health Sciences, Durban University of Technology, Durban, South Africa

**Keywords:** injection in home settings, diabetic patients, knowledge and attitude, sharps waste disposal, healthcare waste management

## Abstract

**Background:**

Sharps waste is hazardous, and it should be disposed of in a proper manner, as it can contribute to transmission of diseases and create a negative impact on the environment. The aim of this investigation was to determine the knowledge, attitudes and practices regarding sharps waste disposal of diabetic patients who inject themselves at home in uMzinyathi District Municipality, a rural area in KwaZulu-Natal, South Africa.

**Methods:**

A quantitative, descriptive cross-sectional study design was adopted using a self-administered questionnaire. Consecutive sampling with a sample size of 308 insulin-dependent diabetic patients from five selected health facilities of uMzinyathi District Municipality was used.

**Results:**

The majority of respondents (62.3%) lacked knowledge regarding proper sharps waste disposal. The vast majority of respondents (90.6%) recognised that sharps waste should be separated from general waste. Among those who acknowledged that someone in their home had been injured by a needle, 53.3% stated that they were motivated to change their method of sharps waste disposal.

**Conclusion:**

The study findings indicated that the majority of the respondents lacked knowledge regarding proper disposal of sharps waste because they were not taught proper methods of sharps waste disposal. There was a general lack of awareness on proper disposal of sharps waste by diabetic patients in the home setting. The study recommended that healthcare workers must place more emphasis on the awareness of proper sharps waste disposal in order to equip diabetic patients with relevant information regarding sharps waste disposal.

## Introduction

The management of chronic diabetes requires regular blood tests and insulin injections in the home setting.^1^ Thousands of items of used sharps waste and potentially infectious waste that are generated every day by diabetic patients within their home environment require proper disposal.^1^ The disposal of sharps waste that is generated in the community has been recognised as an area of public health and environmental health concern.^2^ It is estimated that 7.5 billion syringes and needles are used by diabetic patients, migraines, infertility, arthritis, human immunodeficiency virus (HIV) and other diseases at home every year.^3^ Sharps waste such as needles, disposable scalpels and blades represent 1% of the total waste and are a major source of disease transmission if not properly managed.^1^ Sharps waste disposed of in an improper manner in the community raises concerns, as this waste can put people’s health at risk in terms of injury and contracting blood-borne infectious diseases such as HIV and acquired immune deficiency syndrome (AIDS) and viral hepatitis.^4^

Diabetes affects many people in the world, and 4 million South Africans are estimated to have diabetes and require the use of insulin to manage their condition at home.^3^ Making Medical Injection Safer (MMIS), a Department of Health project, revealed that syringes and needles frequently end up in municipal landfill sites, where municipal workers and the public are exposed to the risk of getting injured and infected.^5^ In the study conducted in South Africa, it was mentioned that studies on proper sharps waste disposal have been conducted globally, but the greatest focus has been on sharps waste disposal in a clinical setting rather than in a home setting.^3^ Disposal of sharps waste from healthcare facilities is extensively regulated, but it is not the same in communities, and yet it poses a public health concern.^2^

Sharps waste disposal among diabetic patients receiving care in home care settings has been found to be unsatisfactory in many countries.^6^ Research conducted in India indicated that less than 10% of cases investigated, specific containers were used to dispose of sharps waste.^6^ In addition, a study conducted in India found that diabetic patients had little knowledge about the risks of improper disposal of sharps waste.^7^

In South Africa, sharps waste management policies and guidelines are available for healthcare facilities.^8^ However, there is nothing similar for hazardous waste generated at home or in the community. The law states that in the health facilities, sharps waste must be disposed of in a sharps waste container that is puncture proof.^5^ Awareness of proper sharps waste disposal is imperative for diabetic patients.

According to the KwaZulu-Natal (KZN) Provincial Department of Health, sharps waste is a form of hazardous waste and 10% – 25% of the total healthcare waste generated by the health facilities is hazardous.^8^ The universal principle, on which the policies and guidelines for waste management in health facilities are based, states that the ‘generator is responsible’ for the management of their waste.^8^ The key stages for waste management include generation, segregation, collection, storage, transportation, treatment and disposal.^8^ It is crucial to follow all these stages when handling waste.^8^

Proper segregation of waste at the point of generation is achieved by using different colour-coded and suitable containers to ensure safe handling and transportation of waste. According to the South African waste classification system, sharps waste is classified as category 4, and it must be stored in a yellow puncture-proof container and must be treated by autoclaving or microwaving before it is disposed of.^8^

There is a gap in knowledge about the disposal of sharps waste among diabetic patients in home settings.^9^ Sometimes sharps waste is found in municipal waste dumps or lying on the ground in living areas.^9^ Improper disposal of sharps waste by diabetic patients at home has become a major problem worldwide.^10^ Patients who receive treatment from the private sector are provided with small sharps waste containers to use at home for disposal of their sharps waste, but patients in the public sector are not provided with these containers.^9^ Patients do not take their used needles back to the clinic but tend to dispose of them in general waste.^9^ As a result, patients, family members, reclaimers and municipal employees who work in waste management are at risk of needle stick injury.^6^

The number of diabetic patients on insulin injections has increased and that creates a high contribution to the waste stream of diabetic products globally.^1^ It has been proven in many studies conducted in different countries worldwide that most diabetic patients who inject insulin dispose of their sharps waste in an improper manner, mostly in domestic waste.^3,9,11,12^ It is imperative to develop sustainable plans to address the issue of sharps waste disposal in the community, especially by diabetic patients.

Education on proper waste disposal, the diseases that can be acquired and other risk factors that can occur through poor waste management must be provided to the community, especially to diabetic patients. Lack of education regarding how and where sharps waste must be disposed of and lack of proper advice from the healthcare workers are the most serious barriers to proper disposal of sharps waste by diabetic patients at home.^13^ A study in developed countries such as the United States found that diabetic patients did not perceive insulin pen needles and lancets as requiring proper disposal methods.^5^ In New Delhi, only 14.1% of diabetic patients received health education on proper disposal of sharps waste from their healthcare givers and the majority (85.9%) of patients were not educated.^5^ Healthcare givers in India either lacked time or were unsure of what to say and how to educate patients; compounding the problem was the shortage of diabetes educators.^5^ In Sri Lanka, 93.0% of diabetic patients reported that they had never received education on how to dispose of their sharps waste.^13^ A Wentworth Hospital study in South Africa concluded that only a few patients who collected their diabetic treatment from the hospital were educated on proper methods of sharps waste disposal. Those who were not educated on proper disposal of sharps waste were disposing them in an improper manner, such as in general waste.^9^

Lack of appropriate storage containers left patients with no choice but to store their sharps waste in their yards, thus making it accessible to children and waste pickers.^4^ Appropriate educations, awareness, knowledge and motivation about sharps waste disposal are the most influential factors in proper sharps waste disposal methods and the management thereof. The aim of this study was to determine the knowledge, attitudes and practices regarding sharps waste disposal of diabetic patients who inject themselves at home in uMzinyathi District Municipality, a rural area in KZN, South Africa.

## Methods and materials

### Study design and site

This quantitative, descriptive cross-sectional study, which investigated the knowledge, attitudes and practices of sharps waste disposal by diabetic patients in home settings, was conducted in five selected primary health care (PHC) facilities in uMzinyathi District Municipality in KZN province of South Africa. The figures from the KwaZulu-Natal Department of Health 2013/2014 annual report show that the district has a high level of poverty, and 88% of the population are uninsured and thus rely on the Public Health Services for healthcare.^14^ The uMzinyathi Health District has four hospitals, one community health centre (CHC), 50 PHC facilities and 12 mobile units.

### Study population and sampling strategy

The study population included diabetic patients who were from 12 years of age (all patients were able to respond to questions), attending the selected facilities and using insulin to manage their diabetes. There were 309 diabetic patients who were sampled using a consecutive sampling strategy, and the researcher sampled 20% of the headcount from participating health facilities. Respondents were selected from five healthcare facilities. These heath facilities were selected because of the high number of insulin-dependent diabetic patients attending those facilities.

#### Inclusion criteria

The following were the study’s inclusion criteria:

- diabetic patients on insulin injections at home
- diabetic patients from 12 years of age and above
- diabetic patients attending selected healthcare facilities in uMzinyathi District Municipality.

#### Exclusion criteria

The following were the study’s exclusion criteria:

- all diabetic patients who were not on insulin injection
- all diabetic patients below the age of 12 years
- diabetic patients receiving care from facilities that were not selected.

### Data collection

Data collection commenced after ethics clearance was obtained from the university and permission was obtained from the Department of Health. Data were collected from June 2019 to July 2019 using a self-administered questionnaire, which was piloted before the commencement of the study. The researcher met the respondents at the PHC facility on their appointment day after the respondents had completed their consultation. This was also done to avoid any disturbances in the flow of work in the clinic.

### Data analysis

Raw data were coded and captured into a Microsoft Excel spreadsheet and analysed using Statistical Package for Social Sciences (SPSS) version 25, where *p* < 0.05 was considered significant. Descriptive statistics, frequency and cross-tabulation were used to analyse the data. Inferential statistical methods such as analysis of variance (ANOVA), chi-square and Fisher’s exact test for categorical data were utilised to test the associations between variables.

### Ethical considerations

The Institutional Research Ethics Committee of Durban University of Technology provided ethical approval for this study (ref. no. REC143/18). Permission was obtained from the KZN Provincial Department of Health Research Committee, the uMzinyathi district health manager and Mpilenhle Medical Centre manager (ref. no. REC 2/18).

The researcher handed out an information letter describing the purpose of the study and how respondents were expected to participate. The researcher verbally addressed queries and questions. Those who agreed to participate signed an informed consent form. An assent consent was provided for respondents younger than 18 years old. Participation in the study was voluntary, and respondents could withdraw from the study at any time without being compromised in any way. The researcher assured respondents of confidentiality throughout data collection and reporting of results. Both the respondents and health facilities were allocated codes for confidentiality.

## Results

### Demographic characteristics

Questionnaires were distributed to 309 participants, and a response rate of 99.7% was achieved. The majority of respondents were women (184, 59.7%) and aged between 13 and 83 years, with a mean age of 53 years. The facility that had the highest number of female respondents (69, 22.4%) was C2 and that with the highest number of male respondents (48, 15.6%) was C3. Fisher’s exact test indicated that the majority of respondents (301, 97.7%) were black South Africans. The level of education ranged from no formal schooling to tertiary education, with 67 (21.8%) having no formal schooling and 51 (16.6%) having tertiary education (Table 1).

**TABLE 1:** Level of education of respondents per facility.

Facility codes	Level of education								Total	
	No formal schooling		Primary school		Secondary school		Tertiary education		*n*	%
	*n*	%	*n*	%	*n*	%	*n*	%		
C1	1	0.3	1	0.3	8	2.6	1	0.3	11	3.6
C2	21	6.8	31	10.1	45	14.6	15	4.9	112	36.4
C3	22	7.1	28	9.1	40	13.0	15	4.9	105	34.1
C4	23	7.5	10	3.2	24	7.8	16	5.2	73	23.7
C5	0	0.0	0	0.0	3	1.0	4	1.3	7	2.3

**Total**	**67**	**21.8**	**70**	**22.7**	**120**	**39.0**	**51**	**16.6**	**308**	**100.0**

Note: Chi-square test = 0.009.

The shortest duration of living with type 2 diabetes mellitus was six months, while the longest duration was 83 years. A few of the respondents (20, 6.5%) did not disclose the duration of living with type 2 diabetes mellitus. A substantial number (157, 51.0%) of respondents across the five facilities used syringes and needles to inject themselves (*p* ˂ 0.05), and a few used lancet pens to inject themselves.

### Practices on sharps waste disposal

The majority of the respondents in this study improperly disposed of sharps waste directly into general waste bins, into the toilet, via burning and using special containers such as a 2-L plastic container, which they put in general waste (Table 2). They were further asked if they had been accidentally pricked by their insulin needles after throwing them away, to which only 58 (18.7%) stated that they had been pricked.

**TABLE 2:** Method of disposing sharps waste by respondents from all facilities.

Method of disposing	C1	C2	C3	C4	C5	Frequency	%
Directly into the general waste bin	6	43	13	26	4	92	29.8
Puncture-resistant container thrown into the dustbin	2	0	0	4	0	6	1.9
Puncture container then taken to the clinic	0	0	0	2	0	2	0.6
Put in plastic and then thrown in general waste	1	0	0	1	1	3	0.9
Sewage system or toilet	2	48	70	28	1	149	48.3
Burnt	0	16	18	11	0	45	14.6
Buried	0	3	2	0	0	5	1.6
Juice bottle or 2-L plastic bottle	0	2	0	0	0	2	0.6
Thrown in the river	0	2	0	0	0	0	0.6
Burnt and then thrown in the toilet	0	0	0	2	1	3	0.9
Wrapped with paper and then thrown in general waste	0	0	0	1	0	1	0.3

In order to gauge the respondents’ knowledge of health risks associated with sharps waste, respondents were asked about the risks associated with sharps waste. The majority (237, 76.9%) of respondents agreed that contracting diseases was the common risk (Table 3).

**TABLE 3:** Respondents’ perceptions of the risks associated with sharps waste.

Risks associated with sharps waste	Number of respondents	%
Self injury	2	0.6
Children play with needles	13	4.2
Someone could get hurt	3	0.9
Diseases	240	77.9
Getting injured	6	1.9
Not aware of any	44	14.2

**Total**	**308**	**100.0**

### Knowledge of and attitude towards sharps waste disposal

When assessing whether respondents were educated regarding sharps waste disposal, the majority of respondents (193, 62.6%) in all five facilities indicated that they lacked knowledge about proper disposal of sharps waste and they were not educated in this regard. Fewer respondents (26, 8.4%) were afraid to personally ask their doctors or nurses about proper disposal of sharps waste. The respondents were asked: ‘Do you believe that improper sharps waste disposal is a serious problem?’ The majority (248, 81.0%) of respondents agreed that improper sharps waste disposal was a serious problem. The vast majority (279, 90.6%) of respondents agreed that it was necessary to separate sharps waste from general waste. In addition, 240 (77.9%) respondents had knowledge of the health risks associated with sharps waste, and the same number of respondents were knowledgeable that contracting diseases was the most common risk associated with sharps waste. The respondents were asked if they would participate in a programme where they could drop off their needles back at the facility, to which the overwhelming majority (296, 96.1%) stated that they would consider this option on the condition that it was free.

The respondents were further asked whether their used insulin needles had accidentally pricked anyone in their home after they were thrown away. The majority (269, 84.4%) of respondents stated that no one had been pricked, while some (48, 14.2%) said that someone other than themselves had been pricked by the sharps waste that was thrown away after use. Respondents who indicated that someone from their homes was pricked by a used sharp waste were asked whether the incident had prompted them to change how they disposed of their sharps waste. The majority of respondents (27, 53.3%) indicated that they were not motivated to change their method of sharps waste disposal.

## Discussion

In this study, women were in the majority, and this was similar to a study conducted in the United Arab Emirates on assessment of awareness of diabetic patients regarding safe disposable of their insulin syringes, where women were in the majority (81, 54.0%).^3^ Women are more prone to contracting diseases than men because of their deoxyribonucleic acid (DNA).^15^ Furthermore, women are more inactive but put an effort into eating healthily.^16^ The current study indicated that respondents with a secondary level of education constituted a significant majority (*p* ˂ 0.05); however, they still disposed of sharps waste improperly, to the same extent of those with lower levels of education. This study clearly indicates that the demographic data of respondents, such as age, gender and the level of education, were not associated with proper disposal of sharps waste. This was found to be similar to a study in the United States, where age, gender and education were not associated with proper disposal of sharps waste.

Proper disposal of sharps waste is very important because doing so will minimise the negative effects on the health of the community and the environment. The majority of the participants in this study improperly disposed of sharps waste directly into general waste bins, using different types of containers. A study conducted among diabetic patients found that the proportion of various kinds of sharps waste disposed in general waste varied from 46.9% to 67.6%.^6^ In Ethiopia and Sri Lanka, it was reported that the highest number of respondents disposed of their waste in general waste bins.^12,15^

Incorrect sharps waste disposal can lead to needle stick injuries of diabetic patients, their families and community members, as some respondents reported a needle stick injury to either themselves or family members. This could increase the risk of transmission of blood-borne diseases. Because of these incidences of needle stick injury, some respondents reported that they changed their behaviour regarding how they disposed of their sharps waste. Similarly, in Malaysia, a few respondents admitted that they had sustained needle stick injuries after using needles, as had their family members.^17^ When waste is disposed of in an improper manner, it could have a negative effect on the environment, causing land and water pollution. When sharps waste is disposed of incorrectly in the community, it can increase the transmission of diseases to children, who can take used needles to play with. In addition, sharps waste can have a negative impact on human health if it is not properly disposed of, including serious diseases such as HIV and AIDS and hepatitis.^18^ As such, in South Africa, 48 children were treated with azidothymidine (AZT) in a hospital after being pricked with needles that were found in a field in the area of Elsie’s River in the Western Cape.^19^ The attitude of diabetic patients towards disposal of sharps waste is crucial; it could mould their behaviour towards proper waste management in their homes.

From the results of this study, it is evident that the majority of respondents disposed of their sharps waste in an improper manner. Most of these respondents disposed of their sharps waste in general waste. This can have a negative effect on human beings and the environment around them. This was consistent with the findings in a South African hospital, where less than 4% of diabetic patients were educated on proper sharps waste disposal. Most of them disposed of their sharps waste in general waste.^9^ In a study in India, respondents had no knowledge about proper disposal of sharps waste.^9^ To achieve the goal of proper sharps waste disposal in the home setting by diabetic patients, health education is the most crucial point.

Regarding respondents’ knowledge of proper disposal of sharps waste, 81.0% of respondents knew that improper sharps waste disposal is a serious problem which could cause harm to the environment and human beings. Across all the five facilities, 77.9% of respondents reported knowledge that contracting diseases was a common risk associated with improper sharps waste disposal. Respondents knew this from their general knowledge, as they were not educated about this by the healthcare workers. However, in the current study it was noted that the majority of respondents were not educated on proper disposal of sharps waste. There are key stages of cradle to grave in waste management that are very important and are interrelated. These stages are segregation, collection, storage, transportation, treatment and disposal.^8^ Proper sharps waste disposal is one of these stages; therefore, respondents were asked questions on the method they used to dispose of their sharps waste. The findings indicate that the majority of the respondents disposed of their sharps waste directly into general waste bins or flushed them down the toilet. Of all the respondents, only two reported to have taken the sharps waste to the clinic on the next scheduled clinic visit. These findings proved that there is a serious problem with current practices of sharps waste disposal by diabetic patients at home.

In a study conducted in Sri Lanka, the majority of respondents reported to be aware of blood-borne infections, which they associated with sharps waste.^13^ Contrary to this finding, in the north-east peninsula of Malaysia, respondents reported that they were not aware of any blood-borne infections associated with being pricked by a used needle.^17^ Nurses and doctors have a good platform to inform the diabetic patients about the importance of proper sharps waste disposal.

In the current study, it was proven that there is a lack of providing education on sharps waste disposal by healthcare providers to the majority of respondents. Similar results from a Sri Lankan study found that the majority of respondents were not educated on proper sharps waste disposal.^13^ Only a few respondents in the current study were educated by nurses and doctors. Some respondents learnt from television shows and verbally via community caregivers (CCGs), waste officers and friends. Similarly, in Ethiopia, the majority of respondents were educated by doctors and nurses.^20^ Currently, there are no needle care educational programmes taking place within the uMzinyathi District Municipality. Because of financial implications and lack of knowledge, respondents had failed to implement the desired practice of proper waste disposal.

The finding of the current study was that there was little emphasis in the clinics on educating patients regarding proper disposal of sharps waste at home, with the majority reporting that they were not educated in their healthcare facilities. Respondents lacked information on proper disposal of sharps waste, even though they were aware that sharps waste is associated with transmission of diseases. The majority of respondents disposed of their sharps waste directly into general waste or flushed them down the toilet. From the investigation, it can be surmised that although some of the respondents have been educated regarding sharps waste disposal, the majority are still ignorant regarding the best way to dispose of their used insulin needles.

### Limitations of the study

This study was limited to the chosen setting without including the larger part of the province and other provinces. There is a paucity of information on this topic, particularly in South Africa. The KZN Department of Health has stopped the use of registers for chronic patients, and this made it difficult to identify diabetic patients.

There is a need to conduct similar studies in other areas of the province and in the country as a whole so that more diabetic patients from other population groups can be engaged. Further research will shed light on focus areas, thereby suggesting the best way of managing the disposal of sharps waste in the community. Further research can assist in closing the gap between the healthcare workers and diabetic patients, presenting new ways or tools to be used by nurses, doctors and CCGs when they are conducting health education with patients.

### Recommendations

Healthcare workers need to place more emphasis on the awareness of proper sharps waste disposal in order to equip diabetic patients with relevant information regarding sharps waste disposal, because healthcare workers are on the front lines when it comes to educating diabetic patients. Healthcare workers need support from community and nonprofit organisations to educate the community. Education of the patients on health risks associated with sharps waste and other negative impacts of improper sharps waste disposal is imperative.

Having programmes in the communities to assist diabetic patients with the disposal of sharps waste could play a major role in the proper disposal of sharps waste. Lack of education on proper sharps waste disposal for diabetic patients is a contributing factor to the practice of improper sharps waste disposal. Formulation of support groups and programmes on proper disposal are needed in the communities, as these have proven to be successful in other countries.

Programmes such as Bring Back Waste to the Facility, where diabetic patients bring back their used needles and lancets to the healthcare facility when they return for their follow-up visits, should be implemented. This will minimise the needle stick injuries and the transmission of blood-borne diseases in the community. Standardised educational programmes should include different kinds of media, including informational videos and pamphlets to impart information on proper disposal of sharps waste and to demonstrate practically how sharps waste should be disposed of. These educational materials must be in people’s language for them to understand it.

The use of CCGs as part of PHC re-engineering outreach programmes could also play a vital role in raising awareness of the community regarding proper waste segregation and particularly sharps waste disposal. Community caregivers should be trained with the appropriate knowledge and skills to pass on to the patients and community members when they conduct their outreach programmes.

The use of environmental health practitioners’ expertise in the communities is another recommendation. Environmental health practitioners have a role to play in waste management because of their broad range of core functions on preventive health. Environmental health practitioners assess and control environmental factors that can potentially affect health, to prevent diseases and create supportive environments. They focus on prevention, consultation, investigation and education of the community regarding health risks and maintaining a safe environment. Environmental health practitioners should conduct health education on proper waste management in the community, using their skills in educating people and also using the educational material that is relevant to people and in their home language.

Good governance, compliance and improved healthcare risk waste management practices across the country should be standardised through policies. Government should formulate policies and guidelines regarding compliance on sharps waste disposal in home settings, including public participation in the formulation process. Where possible, diabetics should be provided with small sharps containers to dispose of their needles and lancets, which they would return to the health facility once they are full.

## Conclusion

This study was the first of its kind to be conducted in South Africa in a rural area, focusing on diabetic patients who inject insulin at home. There is generally a lack of awareness on proper disposal of sharps waste by diabetic patients in the home setting. This is a serious matter which requires attention from the healthcare professionals, because they are the ones who are entrusted with the responsibility of providing the diabetic patients with information on proper disposal of sharps waste. The onus also lies with policymakers, who have the responsibility of formulating and enforcing compliance regarding relevant policies and guidelines regulating the risk of healthcare waste in the community.
